# Effects of Live *Saccharomyces cerevisiae* Yeast Administration in Periparturient Dairy Cows

**DOI:** 10.3390/ani14030472

**Published:** 2024-01-31

**Authors:** Lorenzo Benedetti, Luca Cattaneo, Alessandro Vercesi, Erminio Trevisi, Fiorenzo Piccioli-Cappelli

**Affiliations:** Department of Animal Science, Food and Nutrition (DIANA), Faculty of Agricultural, Food and Environmental Sciences, Università Cattolica del Sacro Cuore, 29122 Piacenza, Italy; lorenzo.benedetti1@unicatt.it (L.B.); luca.cattaneo@unicatt.it (L.C.); fiorenzo.piccioli@unicatt.it (F.P.-C.)

**Keywords:** transition period, probiotic, yeast, rumen fermentation

## Abstract

**Simple Summary:**

The transition period is a crucial phase in the life of dairy cows, and the administration of live yeasts could have positive effects on health and milk yield. Live yeast activity in the rumen can increase fiber digestibility and feed intake. In this study, the impact of live yeast supplementation on milk yield and composition, feed intake, rumination time, and metabolic profiles was evaluated. The administration of live yeast resulted in a decreased rumination time paired with an increased milk yield, with a reduced level of reactive oxygen metabolites.

**Abstract:**

Dairy cows face several challenges during the transition period, and the administration of live yeast might be useful to mitigate this stressful condition. In the current study, the effects of live yeast administration on milk production, feed intake, and metabolic and inflammatory conditions were evaluated. Multiparous Holstein cows were enrolled in this randomized controlled trial and received either a control diet (CTR, *n* = 14) or the control diet plus 4 g/d of live *Saccharomyces cerevisiae* yeast (LSC, *n* = 14) from −21 to 56 days relative to calving. Dry matter intake, milk yield and composition, and rumination time were monitored daily. Blood samples were collected at −21, −7, 3, 14, 28, 42, and 56 days relative to calving to evaluate the metabolic profile. Fecal samples were collected at 56 days relative to calving to measure volatile fatty acids and feed digestibility. No differences between groups were observed in dry matter intake. Compared with CTR, rumination time was lower in LSC in after calving. Although there were no differences in milk components between groups, LSC had greater milk yield in the last three weeks of the study than CTR. No differences were observed in inflammatory markers or other plasma metabolites, except for β-hydroxybutyrate, which was higher in LSC, and reactive oxygen metabolites (ROMs), which were lower in LSC. Overall, these outcomes suggest that live yeast supplementation had some positive effects on milk yield and oxidative status.

## 1. Introduction

The transition period is one of the most critical phases in the life of dairy cows, as it is characterized by several metabolic, physiologic, endocrine, and immune adaptations [1,2]. This period includes the last three weeks before and the first three after calving. With the onset of lactation, voluntary feed intake decreases while energy requirement increases, resulting in a negative energy balance [1]. The magnitude of the negative energy balance during the transition period can vary, and it is strongly related to susceptibility to diseases. Moreover, almost all periparturient cows, even the healthy ones, experience inflammation in this phase [3]. The severity of the inflammatory response, which mainly arises from the uterus, the mammary glands, or the gastrointestinal tract, determines the outcomes (i.e., risk of clinical disorders or diseases). Additionally, the drastic change from the fibrous diet of the dry period to the more energy-dense diet of lactation requires structural and functional adaptations in the rumen [4]. The rumen itself plays an important role in the inflammatory and oxidative stress responses, producing proinflammatory mediators through interaction with lymphoid tissues along the gastrointestinal tract [5].

The use of nutraceuticals might represent a practical solution to mitigate the inflammatory response during the transition period, reducing disease incidence in turn [5]. In this context, the use of yeast-based products is receiving growing interest, particularly as a means to reduce antibiotic and antimicrobial use. The most widely used yeast species is *Saccharomyces cerevisiae*, which can be administrated to cattle in live form [6]. Although the exact mode of action of *S. cerevisiae* has yet to be fully elucidated, multiple mechanisms have been proposed. Live *S. cerevisiae* can use ruminal oxygen [7] and promote the action of either cellulolytic or lactate-utilizing bacteria [8], stabilizing rumen pH. Moreover, *S. cerevisiae* contains a mixture of different components (e.g., polysaccharides, oligosaccharides, and oligopeptides) that can influence metabolism and the inflammatory response. In particular, some components of its cell wall, in particular β-glucans, can interact with the host immune cells and prevent the binding of bacteria to the intestinal wall, resulting in an immunomodulatory effect [9]. Several studies demonstrated that live *S. cerevisiae* supplementation can have positive effects on the performance and health of dairy cows [10,11], mainly because of its stabilizing effect on rumen pH. Some authors proposed that the positive effects on rumen pH could be associated with an increased meal frequency with yeast supplementation [12]. Other positive effects of yeast supplementation have been observed on milk yield, dry matter intake, and organic matter (in particular, fiber) digestibility [13]. This could be a consequence of the stabilization of the ruminal environment, which, in turn, protects the animal from diseases occurring at a lower rumen pH as consequence of the increase in concentrates in the diet during the lactation period. 

We hypothesized that inclusion of live *S. cerevisiae* in the diet of periparturient dairy cows could improve feed intake, rumination time, milk yield, and metabolic profiles because of the improvements in rumen function. Thus, the aim of this study was to evaluate the effects of live *S. cerevisiae* yeast supplementation in multiparous Holstein cows during the transition period on feed intake and digestibility, milk yield and composition, rumen activity, and metabolic profiles. 

## 2. Materials and Methods

### 2.1. Animal Management and Experimental Design

This study was carried out at the Università Cattolica del Sacro Cuore experimental farm (Cerzoo, Piacenza, Italy), in accordance with animal welfare guidelines and Italian laws on animal experimentation (DL n. 26, 4 March 2014; Italian Health Ministry authorization n° 694/2022-PR) and ethics. Twenty-one days before their expected calving dates, 28 multiparous Holstein dairy cows were continuously enrolled from September 2022 through November 2022 and divided into two groups, balanced for parity, previous lactation length and milk yield, disease occurrence, and Body Condition Score (BCS). The control group (CTR; *n* = 14) received dry-period and lactation diets typical of Northern Italy, formulated according to the NRC recommendations [14]. The composition and nutritional characteristics of the diets are reported in Table 1. The other group (LSC; *n* = 14) received the same diets supplemented with 4 g/d of live *S. cerevisiae* (Biosprint^®^, Prosol S.p.A., Madone, BG, Italy; strain MUCL 39885; 1.5 × 10^10^ CFU/g of product), mixed with 100 g of wheat middlings, from −21 to 56 days relative to calving (DRC). The dose has been agreed with the manufacturer. The same quantity of middlings was added to the diet of CTR. Yeast and wheat middlings were mixed with the total mixed ration (TMR) in a mixer wagon. At the beginning of the trial (i.e., −21 DRC), cows were moved to a straw-bedded pen where they received the dry-period diet delivered at 0930 h. Cows remained in this pen until calving, when offspring were immediately separated from their dams and weighed. Then, cows were moved to a freestall pen, where they received the lactation diet, delivered at 0830 h. Cows were milked twice daily (0500 and 1700 h). During the study period, regular checks were made and samples were collected according to the schedule described below.

### 2.2. Health Status, Body Weight, Body Condition Score, and Rectal Temperature

Health status was monitored by the farm veterinarian, and all diseases and disorders were registered. After calving, body weight (BW) was measured automatically with an electronic scale (Afimilk Ltd., Kibbutz Afikim, Israel) at the exit of the milking parlor. The BCS was evaluated on a scale of 1 to 4 points [15], and rectal temperature was measured with a digital thermometer at −21, −7, 3, 14, 28, 42, and 56 DRC by the same trained operator. 

### 2.3. Milk Yield and Composition

Milk yield was recorded automatically at each milking in the parlor and expressed as weekly average. Colostrum was collected for IgG determination using a bovine IgG ELISA kit (E11-118; Bethyl Laboratories Inc., Montgomery, TX, USA). Samples were analyzed in duplicate on a single plate, and the intra-assay CV was 7.05%. Milk samples were also collected at 14, 28, 42, and 56 DRC during the morning milking for the determination of milk composition and somatic cell count (SCC). Milk composition (fat, protein, lactose, casein, and urea) was determined using a Milkoscan FT120 analyzer (Foss Analytics, Hillerød, Denmark), and SCC was determined with a Fossomatic 180 (Foss Analytics, Hillerød, Denmark). Then, the Somatic Cell Score (SCS) was calculated [16].

### 2.4. Feed Intake, Rumination Time, and Fecal Samples 

In both pens, cows were fed through the Roughage Intake Control (RIC, Hokofarm Group, The Netherlands) system, which allows monitoring of daily TMR intake. Rumination time was measured daily with the AfiCollar system (Afimilk Ltd., Kibbutz Afikim, Israel) from −21 to 56 DRC. Daily DMI and rumination data were condensed to weekly averages.

Fecal samples were collected manually in plastic bags from the rectal ampulla at 53, 54, 55, and 56 DRC at different times during the day (0800 h at 53 DFC, 1100 h at 54 DRC, 1600 h at 55 DRC, and 2100 h at 56 DRC), and then 150 g from each sample was pooled to obtain a representative sample of a whole day. In 200 g of freshly pooled samples, DM was determined after drying for 168 h in a ventilated oven at 65 °C. Then, samples were milled using a laboratory mill with a 1 mm screen; ADL content was obtained according to the method described by Van Soest et al. [17] and used as a marker of digestibility of TMR. The analyses were carried out with the Ankom Daisy incubator (ANKOM Technology, Macedon, NY, USA). For VFA analysis, 10 g of each freshly pooled fecal sample was added to 90 mL of distilled water and homogenized for 3 min in a stomacher (BagMixer 400, Interscience International, Saint Nom la Bretêche, France). After filtration through eight-layer medical gauze, aliquots were stored at −20 °C before analysis. Concentrations of fecal VFAs were analyzed with a gas chromatograph (model 7820A; Agilent Technologies, Santa Clara, CA, USA) as described by Minuti et al. [18].

### 2.5. Blood Samples and Immunometabolic Profile

Blood samples were collected before the morning feed delivery (08:00 a.m.) from the jugular vein into heparinized tubes (Vacumed, FL medical srl, Torreglia (PD), Italy) at −21, −7, 3, 14, 28, 42, and 56 DRC. Blood samples were immediately cooled in a mixture of water and ice. An aliquot of blood was centrifuged for 10 min to determine the hematocrit, and the remaining sample was centrifuged at 3500× *g* for 16 min at 6 °C to collect plasma, which was stored at −20 °C for subsequent analyses. Plasma was analyzed using a clinical autoanalyzer (ILAB 650; Werfen-Instrumentation Laboratory, Milan, Italy). Particularly, Ca, P, Mg, Zn, Na, K, Cl, glucose, cholesterol, urea, ceruloplasmin, total protein, albumin, globulin, aspartate aminotransferase (AST-GOT), y-glutamyl transferase (GGT), alkaline phosphatase, bilirubin, haptoglobin, nonesterified fatty acids (NEFA), β-hydroxybutyrate (BHB), paraoxonase, myeloperoxidase, thiol groups, ferric reducing antioxidant power (FRAP), total reactive oxygen metabolites (ROMs), and advanced oxidation protein products (AOPP) were analyzed as described previously [19,20]. 

### 2.6. Statistical Analysis

Data were analyzed with SAS software (release 9.4, SAS Institute Inc., Cary, NC, USA). The normality of the distribution of data was checked with the UNIVARIATE procedure. Outliers were visually assessed, and rarely was more than one data point per variable removed. Data about milk, feeding, rumination behavior, and plasma biomarkers were analyzed with repeated-measures mixed models using the GLIMMIX procedure. The statistical models included the fixed effects of treatment (Trt; CTR vs. LSC), time (T; days or weeks relative to calving), and their interaction (Trt × T) and the random effect of the cow. Denominator degrees of freedom were estimated with the Kenward–Roger method. The covariance structure (among compound symmetry, autoregressive, Toeplitz, and spatial power) with the lowest Akaike information criterion was included in the models. Data obtained before and after calving were analyzed separately. In BW and BCS models, values measured at enrollment were included as a covariate. Pairwise comparisons were performed with the least significant difference test. Data about calf birthweight were analyzed with a mixed model, considering the fixed effects of treatment and sex of the calf. Statistical significance was defined by *p* ≤ 0.05, and tendencies were defined by 0.05 < *p* ≤ 0.10. 

## 3. Results

### 3.1. Health Status, Body Weight, BCS, and Rectal Temperature

Before the enrollment, groups were balanced overall, with no significant differences in lactation number, BW, BCS, cumulative milk yield or days in milk in the previous lactation, or milk yield at dry-off (Table 2).

During the whole study period, no clinical disease was detected. No differences were noted in BW (Table 3) or BCS between the two groups (Table 3). Calf birthweight was not different between groups (45.6 vs. 42.6 ± 1.58 kg, respectively, for LSC and CTR, *p* = 0.17). Overall, there were no differences between groups in rectal temperature (38.84 vs. 38.79 ± 0.05 °C, respectively, in LSC and CTR, *p* = 0.41; Table 3). 

### 3.2. Milk Production and Composition

**Figure 1:**
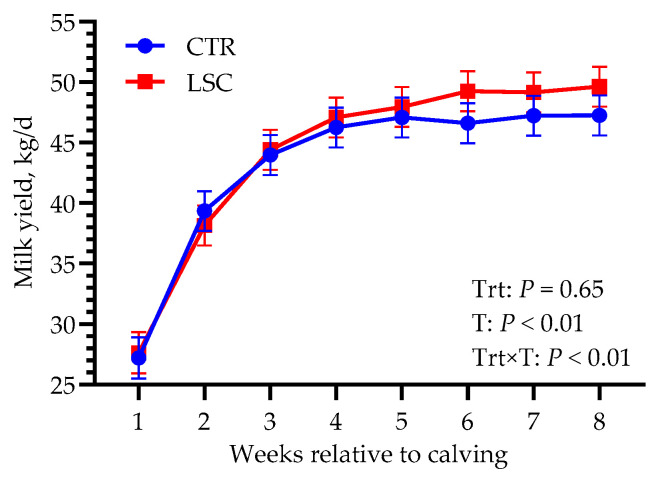
Least-squares means of milk yield in the first 8 weeks relative to calving in Holstein dairy cows supplemented from −21 to 56 days relative to calving with live *Saccharomyces cerevisiae* (LSC) or not (CTR). *p*-values of the main effects of the models are reported (Trt = treatment, T = time, Trt × T = interaction between treatment and time).

Average milk yield in the first 8 weeks of lactation is reported in Figure 1. Although there was no difference overall between groups (43.1 vs. 44.2 kg/d; *p* = 0.65), in the last 3 weeks of the study (i.e., weeks 6, 7, and 8 after calving), milk yield was greater, albeit not significantly, in LSC (on average, ~49 vs. 47 kg/d in LSC and CTR, respectively), resulting in a significant Trt × T interaction effect (*p* < 0.01). No differences between groups were observed in milk composition or SCC (Table 4). However, colostrum IgG content tended to be greater in LSC than in CTR (*p* = 0.09).

### 3.3. Feeding, Rumination Time, and Feces

**Figure 2:**
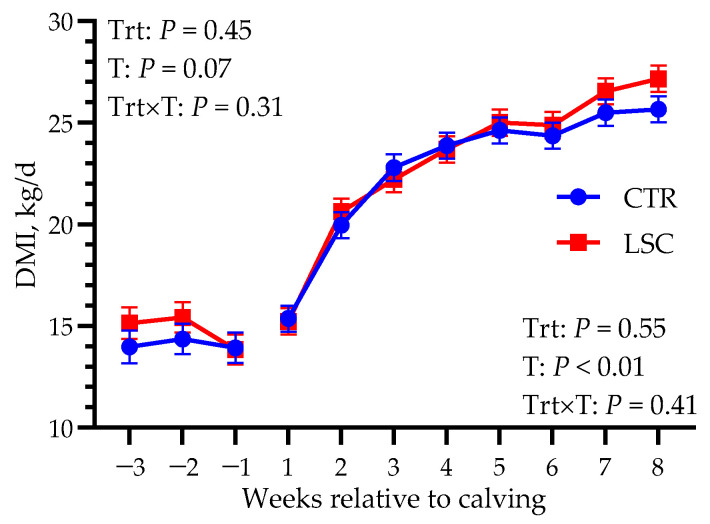
Least-squares means of dry matter intake (DMI) in multiparous Holstein dairy cows supplemented from −21 to 56 days relative to calving with live *Saccharomyces cerevisiae* (LSC) or not (CTR). Data obtained before and after calving were analyzed separately. *p*-values of the main effects of the models are reported (Trt = treatment, T = time, Trt × T = interaction between treatment and time).

**Figure 3:**
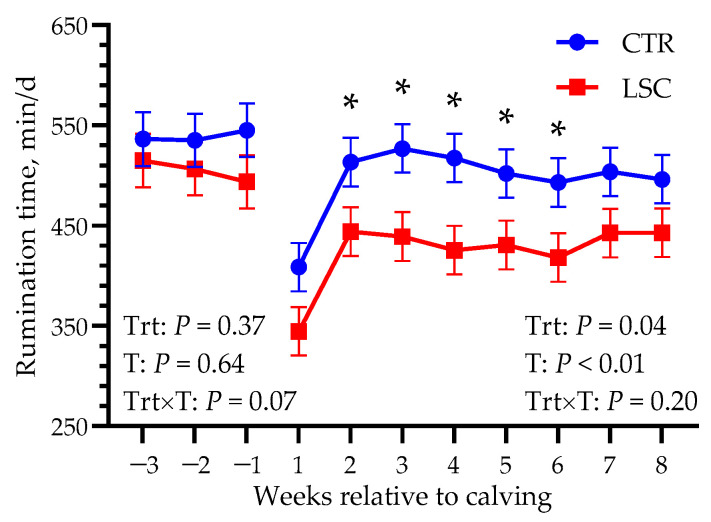
Least-squares means of rumination time in multiparous Holstein dairy supplemented from −21 to 56 days relative to calving with live *Saccharomyces cerevisiae* (LSC) or not (CTR). Data obtained before and after calving were analyzed separately. *p*-values of the main effects of the models are reported (Trt = treatment, T = time, Trt × T = interaction between treatment and time). Significant differences between groups at each time point (*p* < 0.05) are denoted with an asterisk.

No differences in DMI were observed during the whole study period between groups (14.1 vs. 14.8 ± 0.66 kg/d before calving and 22.9 vs. 23.3 ± 0.47 kg/d after calving; *p* ≥ 0.45; Figure 2). Although overall rumination time was not significantly different before calving (539 vs. 505 ± 26.1 min/d; *p* = 0.37), it tended to have different trends between groups, with CTR cows slightly increasing their rumination time the week before calving and LSC slightly decreasing theirs (Trt × T; *p* = 0.07). Moreover, it was different between groups after calving, with greater values in CTR than in LSC (495 vs. 424 ± 23.1 min/d, *p* = 0.04), as shown in Figure 3.

Fecal DM and VFA proportions did not differ between groups (Table 5), and the same was true of fecal ADL, which served as a marker of feed digestibility (11.0 vs. 12.1 ± 0.52% in LSC and CTR, respectively, *p* = 0.13).

### 3.4. Blood Biomarkers

**Figure 4:**
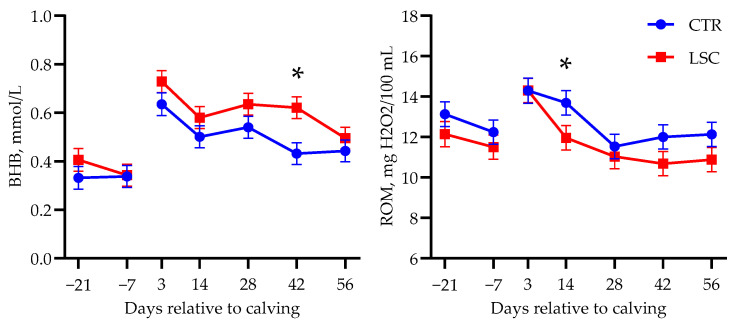
Least-squares means of temporal changes in plasma concentrations of β-hydroxybutyrate (BHB) and reactive oxygen metabolites (ROMs) in multiparous Holstein dairy cows supplemented with live *Saccharomyces cerevisiae* (LSC) or not (CTR) from −21 to 56 days relative to calving. Data obtained before and after calving were analyzed separately. Significant differences between groups at each time point (*p* < 0.05) are denoted with an asterisk.

Table 6 shows the least-squares means of plasma biomarker concentrations measured throughout the study. Packed cell volume did not differ between groups. Overall, energy and protein metabolism markers (glucose, NEFA, urea, and creatinine) did not show any difference, but BHB was higher after calving in LSC than in CTR (Trt; *p* = 0.05), as reported in Figure 4. 

There were no significant differences in inflammation or oxidative stress markers, except for a faster decrease in ROM concentrations after calving in LSC cows (Trt × T, *p* < 0.01). Particularly, at 14 DRC, plasma ROMs were lower in LSC than in CTR (13.7 vs. 12.0 ± 0.60 mg H_2_O_2_/100 mL in CTR and LSC, *p* = 0.04; Figure 4). Before calving, Ca tended to be lower in CTR (*p* = 0.07) and P tended to have a different trend between treatments (Trt × T, *p* = 0.07) because of a slight but nonsignificant decrease in plasma P in LSC at −7 DRC. No other differences between groups in liver function markers or minerals were detected.

## 4. Discussion

Adaptation to the metabolic imbalances that take place during the transition period plays a fundamental role in determining the success of the future lactation and, overall, in the productive career of dairy cows. The administration of live yeast in this critical phase can result in improved performances, supported by improved rumen functionality, as a consequence of the modulation of rumen fermentation [10,11]. In fact, *S. cerevisiae* activity promotes fiber degradation by cellulolytic bacteria, stimulating their adhesion to cellulose [21]. Moreover, it has been shown that, in vitro, *S. cerevisiae* can compete with *Streptococcus bovis*, a lactate-producing bacteria, for the available sugars, causing a decline in the lactate concentration and the stabilization of ruminal pH [22]. At the same time, it promotes the activity of lactate-utilizing bacteria [23]. These actions could, in turn, improve fiber degradation and feed intake. If DMI is increased, positive effects on milk production are expected [24].

In the present study, however, effects of yeast on DMI were lacking. On the contrary, rumination time was surprisingly reduced in LSC cows after calving. With live yeast administration, positive effects on DMI and rumination time would be expected [25] because of the effects of *S. cerevisiae* activity in the rumen described above. However, in agreement with our study, other authors did not find effects on DMI [12,26]. Nevertheless, a possible explanation for the lower rumination time recorded after calving (with a similar DMI) might be related to faster degradability of fiber, usually promoted by yeast activity [27], which could have resulted in a reduced retention time and increased ruminal passage rate [28]. Unfortunately, in the current experiment, rumen fluid samples were not collected to characterize the rumen fermentation pattern; thus information, to confirm this speculation is lacking. Moreover, no difference was noted in our study in fecal ADL or VFA proportions, used as markers of feed digestibility and hindgut fermentation, respectively. Therefore, the reason for the difference observed in rumination time remains to be elucidated.

Despite the lack of effects on DMI, a different pattern in the lactation curve was observed, with increasing milk yield in LSC cows at the end of the last phase of the experimental period (second month of lactation) compared with a rather constant yield in CTR. Despite the greater concentration of blood BHB observed in LSC in the whole lactation period considered, this outcome could not be explained by an increased mobilization of the body fat reserves. Body weight and BCS variations during the period investigated were similar between groups and unaltered by yeast supplementation, and the same was true of plasma NEFA concentrations (also considering the lack of differences in liver function). Additionally, no cases of clinical ketosis were observed in either group. Thus, the reason for the greater plasma concentration of BHB, which always fluctuated within physiological ranges (0.4–0.7 mmol/L), is not clear. Yuan et al. [29] found similar results when supplementing the diet of cows with a yeast product during transition. The increase in plasma BHB might be explained by greater synthesis of ruminal butyrate, which is transformed into BHB during absorption from the ruminal epithelium [30]. Nevertheless, it is not clear whether the activity of live *S. cerevisiae* can increase the ruminal butyrate concentration. The greater blood BHB observed at 42 DRC could also have been associated with the numerically greater milk yield recorded in this period. In this stage of lactation (i.e., after the first month of lactation), considering the unaltered DMI and plasma glucose (as a marker of energy availability), BHB concentrations in those ranges are not pathological and can, indeed, support lactation [31]. 

The postpartum inflammatory response was comparable between the two groups, although LSC cows showed a faster decrease of the concentration of ROMs after calving. This could suggest better modulation of the inflammatory response [21]. Components of yeast cell walls (specifically, β-glucans) can have an immunomodulatory effect [32], likely mediated by the rumen epithelium, and supplementation with *S. cerevisiae* improved the inflammatory conditions in transition-period cows in another study [33]. Cows involved in the trial did not experience any disease or metabolic disorder, as also supported by the body temperature values, which never reached the critical thresholds suggesting potential issues. Indicators of the innate immune response (acute phase proteins and myeloperoxidase) showed similar trends in both groups. Indeed, at the same time, cows treated with yeast showed a lower oxidative stress response, i.e., a lower level of ROMs paired with unchanged levels of available antioxidants (i.e., FRAP) [21]. The greater Ca level observed in the plasma of LSC cows before calving might be related to the numerically different DMI [34], whereas the differences in plasma P are unclear. However, the magnitude of these changes was very limited and unlikely to cause relevant biological effects, as supported by the similar blood concentrations of these minerals after calving and the lack of milk fever cases.

Overall, the effects of live *S. cerevisiae* supplementation in the present study were limited compared with those observed in the literature [6]. There could be two possible reasons for this difference. First, the dose of live yeast used in our study (6 × 10^10^ CFU/d) was smaller compared with those used in other studies (for instance, 10 × 10^10^ CFU/d in the study by Cattaneo et al. [33]). The dose and strain of the yeast used and the productivity, physiological condition, and diet of the cows can affect the animals’ responses to supplementation [35]. Second, the experiment was carried out on an experimental farm, where cows are constantly monitored and raised with high welfare standards. This is supported by the concentrations of the biomarkers related to the inflammatory response and oxidative stress, which were within the reference ranges proposed for the transition period [19] and indicate overall limited peripartum inflammation. The intensity of the inflammatory response was related to the physiological adaptations occurring during the transition period [36]. Considering that the benefits of yeasts might be greater in stressful conditions [37,38], the mild periparturient stress experienced by these cows might have mitigated the observed results. In stressful conditions, the effects of yeasts are usually greater, as cows consistently reduce DMI, and animals supplemented with live yeast can better cope with this condition thanks to the yeast-related improvements in feed digestibility and rumen function [38], as well as the attenuation of immunometabolic pressure. Based on the latter, further studies on this product carried out in different settings could show different outcomes.

## 5. Conclusions

Supplementation with live *S. cerevisiae* from −21 DFC to 56 DFC in multiparous dairy cows increased milk production in the last three weeks of supplementation. Nevertheless, no differences in DMI were noted in supplemented cows, and their rumination time was lower after calving. Although liver function and fat mobilization were unaltered, plasma BHB concentrations were greater in yeast-supplemented cows. Only minor differences in inflammation and oxidative stress markers were found. Interestingly, supplemented cows experienced lower oxidative stress, as supported by a reduced plasma ROM concentration after calving.

Some positive effects on performance and inflammation following the administration of yeast in the peripartum period were confirmed in this study, although the dose of yeast used was lower than in other studies, and this experiment was performed on a farm with high hygienic standards and animal welfare conditions.

## Figures and Tables

**Table 1:** Ingredients and chemical composition of the experimental diets fed during dry and lactation periods.

	Dry Period	Lactation
Ingredient, % DM		
Corn silage	22.94	48.22
Soybean meal	7.96	15.62
Corn meal	-	12.73
Alfalfa hay	-	9.63
Barley silage	35.32	7.44
Crushed barley grain	-	3.64
Mineral supplement ^1^	0.48	1.90
Hydrogenated vegetable fat	-	0.83
Wheat straw	26.55	-
Sunflower meal	6.86	-
Chemical composition ^2^		
DM, %	44.84	50.42
NEL Mcal/Kg of DM	1.26	1.55
Starch, % DM	12.88	27
Sugar, % DM	1.56	2.43
CP, % DM	12.11	16.11
MP, % DM	8.29	10.90
Ether extract, % DM	2.59	3.34
NDF, % DM	52.90	34.04
ADF, % DM	32.44	19.79
ADL, % DM	6.97	2.97
Ca, % DM	0.31	0.72
P, % DM	0.34	0.43
Mg, % DM	0.22	0.24
K, % DM	0.94	1.04
Na, % DM	0.07	0.25
DCAD, mEq/kg DM	250	-

^1^ For dry cows, the supplement contained the following (in descending order): dicalcium phosphate, magnesium oxide, monocalcium phosphate, sodium chloride, and wheat middlings; vitamins—vitamin A 1,500,000 IU/kg, vitamin D3 150,000 IU/kg, and vitamin E 7000 mg/kg. The composition (on a DM basis) was as follows: 11.5% Ca, 8.6% P, 7.5% Mg, and 2.7% Na. For lactating cows, the supplement contained the following (in descending order): sodium bicarbonate, calcium carbonate, monocalcium phosphate, sodium tripolyphosphate, magnesium oxide, magnesium carbonate, sodium chloride, and barley meal; vitamins—vitamin A 900,000 IU/kg, vitamin D3 150,000 IU/kg, and vitamin E 3000 mg/kg. The composition (on a DM basis) was as follows: 11.8% Ca, 10.8% Na, 6.5% P, and 4.5% Mg. ^2^ Calculated according to NRC [14]; DM is dry matter, CP is crude protein, MP is metabolizable protein, NDF is neutral detergent fiber, ADF is acid detergent fiber, ADL is acid detergent lignin, and NEL is net energy for lactation.

**Table 2:** Least-squares means of main characteristics of cows before enrollment.

	Trt			
Item, Unit	CTR	LSC	SEM ^1^	*p*-Value
Lactation number, *n*	2.07	2.29	0.39	0.70
Body weight at dry-off, kg	658	694	18.0	0.16
BCS ^2^	2.89	3.05	0.07	0.12
Previous lactation cumulative milk yield, kg	11,528	11,855	668	0.79
Previous lactation length, d	344	335	10.8	0.57
Milk yield at dry-off, kg/d	11.6	11.3	1.21	0.86

^1^ Greatest standard error of the mean (SEM). ^2^ Evaluated at −21 days relative to calving.

**Table 3:** Least-squares means of rectal temperature, body weight, and BCS in Holstein dairy cows supplemented from −21 to 56 days relative to calving with live *Saccharomyces cerevisiae* (LSC) or not (CTR).

	Trt			*p*-Values ^1^		
Item, Unit	CTR	LSC	SEM ^2^	Trt	T	Trt × T
Before calving						
Body weight ^3^, kg	-	-	-	-	-	-
BCS	3.03	3.09	0.03	0.17	0.94	0.44
Rectal temperature, °C	38.75	38.81	0.06	0.48	0.18	0.90
After calving						
Body weight, kg	634	638	4.78	0.60	<0.01	0.97
BCS	2.60	2.62	0.04	0.80	<0.01	0.93
Rectal temperature, °C	38.78	38.92	0.06	0.40	<0.01	0.19

^1^ *p*-values of the main effect: treatment (Trt), time (T), and their interaction (Trt × T). ^2^ Greatest standard error of the mean (SEM). ^3^ Body weight was measured only after calving.

**Table 4:** Least-squares means of milk components in the first 8 weeks of lactation and colostrum IgG in multiparous Holstein dairy cows supplemented with live *Saccharomyces cerevisiae* (LSC) or not (CTR) from −21 to 56 days relative to calving.

	Trt			*p*-Value ^1^		
Item, Unit	CTR	LSC	SEM ^2^	Trt	T	Trt × T
Milk fat, %	3.59	3.39	0.14	0.29	0.08	0.71
Milk fat, kg/d	1.64	1.60	0.07	0.67	0.60	0.73
Milk protein, %	3.08	3.01	0.06	0.37	<0.01	0.90
Milk protein, kg/d	1.41	1.42	0.05	0.82	0.03	0.44
Milk casein, %	2.42	2.37	0.04	0.42	<0.01	0.86
Milk casein, kg/d	1.11	1.12	0.04	0.84	0.02	0.46
Milk lactose, %	4.75	4.77	0.05	0.72	0.99	0.34
Milk urea, mg/dL	33.2	36.0	3.04	0.52	0.60	0.57
Milk SCS ^3^, *n*	1.00	1.03	0.35	0.97	0.52	0.24
Colostrum IgG, mg/mL	191	219	11.6	0.09	–	–

^1^ *p*-values of the main effects: treatment (Trt), time (T), and their interaction (Trt × T). ^2^ Greatest standard error of the mean (SEM). ^3^ SCS = Somatic Cell Score [16].

**Table 5:** Least-squares means of dry matter (DM) and volatile fatty acid (VFA) molar proportions in feces collected at the end of the study in multiparous Holstein dairy cows supplemented with live *Saccharomyces cerevisiae* (LSC) or not (CTR) from −21 to 56 days relative to calving.

	Trt			
Item, Unit	CTR	LSC	SEM ^1^	*p*-Value
DM, %	15.2	14.7	0.34	0.34
Acetate, mol/100 mol	73.7	73.4	0.56	0.76
Propionate, mol/100 mol	14.5	14.4	0.23	0.77
Butyrate, mol/100 mol	9.88	10.12	0.45	0.71
Isobutyrate, mol/100 mol	0.14	0.16	0.02	0.55
Valerate, mol/100 mol	1.39	1.27	0.13	0.53
Isovalerate, mol/100 mol	0.68	0.68	0.13	0.99
Acetate/propionate ratio	5.11	5.13	0.11	0.94
(Acetate + butyrate)/propionate ratio	5.80	5.83	0.11	0.87

^1^ Greatest standard error of the mean (SEM).

**Table 6:** Least-squares means of plasma biomarkers in multiparous Holstein dairy cows supplemented with live *Saccharomyces cerevisiae* (LSC) or not (CTR) from −21 to 56 days relative to calving. Data obtained before and after calving were analyzed separately.

	Before Calving						After Calving					
	Trt			*p*-Value ^1^			TRT			*p*-Value ^1^		
Item ^2^, Unit	CTR	LSC	SEM ^3^	Trt	T	Trt × T	CTR	LSC	SEM ^3^	Trt	T	Trt × T
PCV, L/L	0.33	0.32	0.01	0.17	0.36	0.83	0.31	0.31	0.00	0.55	<0.01	0.90
Glucose, mmol/L	4.33	4.39	0.05	0.40	0.65	0.98	3.99	4.03	0.07	0.74	<0.01	0.90
NEFA, mmol/L	0.17	0.13	0.03	0.29	<0.01	0.26	0.40	0.46	0.04	0.28	<0.01	0.64
BHB, mmol/L	0.34	0.37	0.02	0.30	0.26	0.21	0.51	0.61	0.03	0.05	<0.01	0.56
Cholesterol, mmol/L	2.45	2.41	0.12	0.80	<0.01	0.81	4.07	3.83	0.16	0.29	<0.01	0.92
Urea, mmol/L	5.00	5.05	0.26	0.90	0.03	0.99	5.99	6.25	0.29	0.52	<0.01	0.28
Creatinine, µmol/L	97.7	97.8	1.64	0.98	<0.01	0.85	85.4	85.8	1.24	0.85	<0.01	0.68
Haptoglobin, g/L	0.10	0.14	0.04	0.44	0.11	0.66	0.18	0.21	0.03	0.46	<0.01	0.73
Ceruloplasmin, µmol/L	2.38	2.21	0.08	0.15	<0.01	0.13	2.28	2.26	0.11	0.90	<0.01	0.32
Zn, µmol/L	8.16	8.63	0.50	0.52	0.09	0.79	7.48	7.91	0.45	0.50	0.05	0.16
Myeloperoxidase, U/L	376.3	372.5	10.89	0.81	0.02	0.15	372.7	372.6	10.8	1.00	<0.01	0.63
Globulin, g/L	41.8	42.7	1.16	0.59	<0.01	0.73	41.6	42.2	1.13	0.72	<0.01	0.56
Total protein, g/L	78.5	79.5	1.11	0.51	<0.01	0.42	79.3	79.8	1.05	0.73	<0.01	0.16
ROMs, mg H_2_O_2_/100 mL	12.7	11.8	0.48	0.19	0.03	0.60	12.7	11.8	0.50	0.18	<0.01	<0.01
Thiol groups, µmol/L	291.7	297.9	6.43	0.50	0.60	0.18	310.5	318.6	7.45	0.45	0.05	0.22
FRAP, µmol/L	110.0	108.0	1.39	0.33	0.37	0.83	137.3	131.8	2.33	0.11	<0.01	0.42
Bilirubin, µmol/L	0.94	0.60	0.17	0.18	0.01	0.25	2.22	2.20	0.25	0.97	<0.01	0.20
Paraoxonase, U/mL	83.6	87.9	2.93	0.32	<0.01	0.74	84.5	85.6	3.34	0.82	<0.01	0.63
Albumin, g/L	36.7	36.8	0.36	0.81	0.27	0.15	37.7	37.6	0.41	0.94	<0.01	0.17
ALP, U/L	50.8	57.7	4.36	0.27	0.32	0.22	45.8	51.5	3.96	0.32	<0.01	0.66
GOT U/L	82.3	79.5	4.30	0.65	0.11	0.78	94.3	92.1	2.33	0.52	<0.01	0.80
GGT, U/L	22.8	21.1	1.42	0.41	<0.01	0.88	21.9	20.3	0.98	0.26	<0.01	0.29
Ca, mmol/L	2.34	2.39	0.02	0.07	0.12	0.09	2.34	2.33	0.03	0.91	<0.01	0.67
P, mmol/L	2.00	1.94	0.08	0.65	0.88	0.07	1.62	1.73	0.08	0.37	0.40	0.24
Mg, mmol/L	0.91	0.91	0.02	0.98	0.21	0.43	1.00	0.99	0.02	0.79	<0.01	0.98
Na, mmol/L	149.3	148.4	0.53	0.25	<0.01	0.55	146.8	146.6	0.45	0.82	<0.01	0.99
K, mmol/L	4.21	4.09	0.08	0.29	0.08	0.12	4.22	4.10	0.08	0.30	0.36	0.43
Cl, mmol/L	108.5	107.9	0.49	0.39	<0.01	0.18	102.8	102.3	0.57	0.59	<0.01	0.91

^1^ *p*-values of the main effects: treatment (Trt), time (T), and their interaction (Trt × T). ^2^ PCV = packed cell volume; NEFA = nonesterified fatty acids; BHB = β-hydroxybutyrate; ROMs = reactive oxygen metabolites; FRAP = ferric reducing antioxidant power; ALP = alkaline phosphatase; GOT = aspartate aminotransferase; GGT = γ-glutamyl transferase. ^3^ Greatest standard error of the mean (SEM).

## Data Availability

The data presented in this study are available on request from the corresponding author. Data are available with the permission of Prosol S.p.A.
